# Dr. Isaac Ray

**Published:** 1881-05

**Authors:** 


DR. ISAAC RAY, well and widely known as a writer on the treatment of insane patients, died March 31, at his residence in this city, in the 74th year of his age. He was born in Beverly, Maine, and graduated at Bowdoin College in 1827, and studied medicine with Dr. Shattuck, in Boston. He practiced for some time in Portland and Eastport, and then took charge of the Insane Asylum at Augusta. Subsequently he was in charge of the Butler Hospital at Providence, and came to Philadelphia in 1867, since which time he has been engaged as a consulting physician. In July, 1880, he was elected an honorary member of the Medico-Psychological Association of London, and received the diploma a few weeks since. He had been in ill health for twenty-five years previous to his death. He was elected a fellow of the College of Physicians of Philadelphia in 1868.

Dr. Ray was a voluminous writer on medical subjects, and included in his works will be found: "Conversations on Animal Economy," 1829; a treatise on the "Medical Jurisprudence of Insanity," 1838, a work termed by Mr. Cockburn, Attorney-

General of England, as "perhaps the most scientific treatise that the age had produced on the subject of insanity in relation to jurisprudence;" "Homicide—Epilepsy," 1867, a review of the case of G. W. Winnemore, executed for murder at Philadelphia, August 29, 1867. He also contributed papers to the *North American Review*, *American Quarterly Review*, *Christian Examiner*, *American Jurist*, *Law Reporter*, *Boston Medical Magazine*, *American Journal of Insanity*, and the *Atlantic Monthly*. The eleventh edition of his "Medical Jurisprudence" was published in this city in 1860.—*Philadelphia Medical and Surgical Reporter.*

